# Sustained release formulation of an anti-tuberculosis drug based on para-amino salicylic acid-zinc layered hydroxide nanocomposite

**DOI:** 10.1186/1752-153X-7-72

**Published:** 2013-04-20

**Authors:** Bullo Saifullah, Mohd Zobir Hussein, Samer Hasan Hussein-Al-Ali, Palanisamy Arulselvan, Sharida Fakurazi

**Affiliations:** 1 Materials Synthesis and Characterization Laboratory (MSCL), Institute of Advanced Technology (ITMA), Universiti Putra Malaysia, Serdang, Selangor, 43400, Malaysia; 2 Laboratory of Molecular Biomedicine, Institute of Bioscience, Universiti Putra Malaysia, Serdang, Selangor, 43400, Malaysia; 3 Laboratory of Vaccines and Immunotherapeutics, Institute of Bioscience, Universiti Putra Malaysia, Serdang, Selangor, 43400, Malaysia; 4 Faculty of Medicine and Health Sciences, Department of Human Anatomy, Universiti Putra Malaysia, Serdang, Selangor, 43400, Malaysia

**Keywords:** Para-Amino salicylic acid(PAS / 4-ASA), MDR-TB, Zinc layered hydroxide, Nanocomposite, 3T3 cell lines

## Abstract

**Background:**

Tuberculosis (TB), is caused by the bacteria, *Mycobacterium tuberculosis* and its a threat to humans since centuries. Depending on the type of TB, its treatment can last for 6–24 months which is a major cause for patients non-compliance and treatment failure. Many adverse effects are associated with the currently available TB medicines, and there has been no new anti-tuberculosis drug on the market for more than 50 year, as the drug development is very lengthy and budget consuming process.

Development of the biocompatible nano drug delivery systems with the ability to minimize the side effects of the drugs, protection of the drug from enzymatic degradation. And most importantly the drug delivery systems which can deliver the drug at target site would increase the therapeutic efficacy. Nanovehicles with their tendency to release the drug in a sustained manner would result in the bioavalibilty of the drugs in the body for a longer period of time and this would reduce the dosing frequency in drug administration. The biocompatible nanovehicles with the properties like sustained release of drug of the target site, protection of the drug from physio-chemical degradation, reduction in dosing frequency, and prolong bioavailability of drug in the body would result in the shortening of the treatment duration. All of these factors would improve the patient compliance with chemotherapy of TB.

**Result:**

An anti-tuberculosis drug, 4-amino salicylic acid (4-ASA) was successfully intercalated into the interlamellae of zinc layered hydroxide (ZLH) via direct reaction with zinc oxide suspension. The X-ray diffraction patterns and FTIR analyses indicate that the molecule was successfully intercalated into the ZLH interlayer space with an average basal spacing of 24 Å. Furthermore, TGA and DTG results show that the drug 4-ASA is stabilized in the interlayers by electrostatic interaction. The release of 4-ASA from the nanocomposite was found to be in a sustained manner. The nanocomposite treated with normal 3T3 cells shows it reduces cell viability in a dose- and time-dependent manner.

**Conclusions:**

Sustained release formulation of the nanocomposite, 4-ASA intercalated into zinc layered hydroxides, with its ease of preparation, sustained release of the active and less-toxic to the cell is a step forward for a more patient-friendly chemotherapy of Tuberculosis.

## Introduction

Tuberculosis (TB) is one of the transmittable diseases caused by the bacteria, *Mycobacterium tuberculosis*. Despite the technological advancements, tuberculosis is still lethal and the main cause of the human deaths in many countries around the world. According to the latest facts and figure about TB in the Global tuberculosis report 2012, there were about 8.7 millions new cases of TB in 2011 and 1.4 million humans lost their lives. TB is one of the top killers of women, with 300 000 deaths among HIV-negative women and 200 000 deaths among HIV-positive women in 2011 [1].

There are three forms of TB, namely drug susceptible-, multidrug resistant- and extensively resistant-TB. Drug susceptible TB is mainly treated with first line anti-TB drugs such as Rifampin, Isoniazid, Ethambutol and Pyrazinamide, and treatment usually lasts from 6–9 months.

When tuberculosis bacteria develop resistance to at least two most powerful first line drugs Rifampicin (RMP) and Isoniazid (INH), it is called multi-drug resistant tuberculosis (MDR-TB) [2]. MDR-TB patients' treatment can last for 20 months [3]. 4-amino salicylic acid is a bacteriostatic agent and is extremely helpful in controlling the growth of bacteria. It is also very useful in avoiding the development of the resistance of bacteria to the other anti-MDR-TB drugs [4]. MDR-TB patients have to take 4 g dose of 4-ASA twice daily during treatment [4]. As we know that the drug development is a time consuming process and requires a huge amount of budget and no new anti-TB drug has been introduced in the market for almost 50 years, therefore, the logical approach in such circumstances is that we should use the currently available drugs more intelligently and effectively.

4-aminosalicylic acid (4-ASA) was introduced in 1948 in clinical trials and was found to be very effective against TB [5]. However, due to its side effects such as anorexia, epigastria distress, nausea, vomiting, abdominal cramps or diarrhea, it is no longer used for drug susceptible TB, but is used for the multi-drug resistant TB and Controlled release formulation would be very useful to resuce the side effects associated with 4-ASA [6,7]. In order to avoid these side effects associated with 4-ASA, various strategies have been adopted by physicians such as the drug given in the form of buffered tablets, granules, coated tablets and salt of acid. The intrinsic half-life of 4-ASA is 45–60 min. The controlled and targeted release can be the best choice to avoid the side effects and would also increase the intrinsic half life of the drug. The sustained release formulation would not only minimize the side effects but would also result in the shortening of the treatment duration and also the dosing frequency. The reduction in the frequency of the dose administration would enhance the patient compliance to the treatment.

Keeping this idea in mind, we have developed the controlled release formulation of 4-amino salicylic acid (4-ASA) with zinc layered hydroxides.

There have been many attempts made earlier by various scientists in the development of drug delivery systems for anti-tuberculosis drugs. Inhalable microparticles of polylactic acid, mannitol microparticles, glucan particles (GPs) and plant proteins etc., have been developed for the delivery of anti-TB drugs, a good review was written by Saifullah et al. Which discusses many of these delivery systems in detail [8].

Zinc layered hydroxide (ZLH) has the structure similar to that of brucite (Mg (OH) _2_ which is a hydrotalcite-like compound and is formed by a layered unit with the metal cations octahedrally coordinated with hydroxyl groups. A quarter of the octahedral of zinc cation coordination is displaced from the main layer to tetrahedral sites located above and below each empty octahedron and can be represented by the general formula of hydrotalcite, ${\left[M_{1-x}^{2+}M_{x}^{3+}{\left(\mathrm{OH}\right)}_{2}\right]}^{x+}{\left(A^{\text{m}-}\right)}_{x/m}.{\mathrm{nH}}_{2}O$, where M^2+^ is divalent cations, M^3+^ is trivalent compound and A^m-^ is an exchangeable anion, with the charge, (m-) [9].

Characteristics of zinc layered hydroxides are similar to that of layered double hydroxides which have plenty of benefits as a drug delivery system, such as their ease of laboratory preparation with controlled particle size [10]. The drugs can easily be accommodated in the space of interlayers by ion exchange as compared to the other nanoparticles which need surface modification i.e. functionalization of the surface [11,12].

ZLH, have high zeta potential, 20–30 mV like to that of the layered double hydroxides, which is a strong driving force onto the surface of the cell. On the other hand, other inorganic nanoparticles, due to their low solubility, they accumulated in cells. The biocompatibility, controlled release and easier degradation without accumulation in the body are important characteristic of the layered double hydroxides and zinc layered hydroxides, which make them strong candidates as drug delivery systems. In previous studies it has been reported, that the Zn-Al LDH does not show any toxic effect on Chinese hamster ovary cells at low concentration [13]. ZLH has also been used as a drug delivery system of an anticancer compound, ellagic acid [14]. The intercalation of hippuric acid into ZLH has resulted in the less toxic of the compound compared to the pure hippuric acid itself [15]. It was also reported that the controlled release formulation of an antihistamine was obtained by forming the cetirizine-ZLH nanocomposite [16].

In this study, we report a controlled release formulation of 4-amino salicylic acid, an antituberculosis agent by forming a nanocomposite zinc layered hydroxides(4-ASA-ZLH) nanocomposite.

## Experimental

### Materials

Analytical grade chemicals were used without any further purification. 4- amino salicylic acid 99% purity and zinc oxide were purchased from Sigma-Aldrich. Dimethyl sulfoxide (DMSO) was used as solvent and was purchased from Ajax Finechem (Sydney, Australia) with 0.1% water content.

### Synthesis

The drug, 4-aminosalicyclic acid (0.4 mol) was mixed into 50 mL (i.e 25 mL Deionized water and 25 mL DMSO) and was stirrer for 20 minutes at room temperature. About 0.2 g zinc oxide was mixed with 50 ml deionized water and stirred for 25 minutes. The drug solution was added drop wise into zinc oxide suspension, and the pH of the final solution was raised to 7.9 by drop wise addition of 0.5 M sodium hydroxide solution. The solution was kept on stirring for 18 hours at 70°C. The sample was centrifuged and washed with deionized water and kept in an oven at 70°C for 48 hours and the sample was then subjected to further characterizations [14,17,18].

### Controlled release study

The release of 4-aminosalicylic acid was performed on different human body simulated pH 4.8 and 7.4 buffer solutions (0.1 M). The phosphate-buffered solution contains a number of anions, such as Cl^−^, monobasic phosphate H_2_PO_4_^−^, and dibasic phosphate HPO_4_^2−^. About 0.6 mg of the nanocomposite was placed into 3.5 mL of either a pH 4.8 or pH 7.4 buffer solution. The absorbance were measured at *λ*_max_ = 268 nm [19] by a UV–vis absorption spectrophotometer.

To compare the release of 4-aminosalicylic acid from nanocomposite and from a physical mixture of 4-aminosalicylic acid with ZLH, a specific amount of the physical mixture of 4-aminosalicylic and the pristine ZLH was used. The release of the active, 4-aminosalicylic was determined as previously described above.

### Cell culture

Healthy 3T3 mouse fibroblast cells were obtained from ATCC (USA). 3T3 fibroblast cells were cultured at 37°C and 5% CO_2_ in high glucose Dulbecco’s Modified Eagle Medium containing 10% fetal bovine serum, 0.5% penicillin–streptomycin, 1% glutamine, and 1% non-essential amino acids. Cultured cells were passaged using 0.25% trypsin. At 80% confluence, cultured cells were harvested using 0.25% trypsin and were subcultured/seeded in 96-well plates for further characterizations.

### Assessment of cytotoxicity by MTT assay

Cytotoxicity of synthesized nanocomposites was evaluated by MTT assay, where the cell viability is spectroscopically measured as the total mitochondrial activity. For MTT assay, cultured 3T3 cells were seeded and cultured in a 96-well culture plate (at a density of 1 × 10^4^ cells per well) in 100 μL cell culture medium (DMEM with 10% fetal bovine serum) and the same were incubated for 24 hours under cultured conditions at 37°C and 5% CO_2_. Then, cultured cells were washed with phosphate-buffered saline (PBS, pH 7.4) and were then incubated with a medium (100 μL) containing dispersed nanocomposite in various gradient concentrations, range from 0.781 μg/mL to 50 μg/mL. Cells not exposed to nanocomposite were served as control in each experiment. After 24, 48 and 72 hours of incubation, the medium were removed from 96 well plate and incubated with 100 μLof the freshly prepared MTT reagent in DMEM for another 3–4 hours. Observance of the solution in 96 well plates was measured at 570 NM using a microplate reader. Data are expressed as the percentage of cell viability compared with untreated cells under the same experimental conditions.

### Characterization

Shimadzu XRD-6000 diffractometer (Tokyo, Japan) was used to obtain the powder X-ray diffraction (PXRD) patterns. Radiation CuK_α_ at 30 KV and 30 mA was used for recording the PXRD difractograms between 2-60^o^. Fourier transform infrared (FTIR) spectra of the materials were recorded over the range of 400–4000 cm^-1^ on a Perkin- Elmer 100 series spectrophotometer by direct sample method. A LECO, model CHNS-932 instrument (St Joseph, MI) was utilized for the analysis of carbon, hydrogen, nitrogen and sulfur (CHNS). A Mettler Toledo instrument (Greifensee, Switzerland), was used for the thermo gravimetric and differential thermo gravimetric analyses (TGA-DTG) analysis. Micromeritics ASAP 2000 (Norcross, GA), was used for the surface area analysis of the samples, by nitrogen gas adsorption–desorption method at 77 K. For the determination of the morphology of the sample surface, a scanning electron microscope (SEM), JOEL JSM-6400 (Tokyo, Japan) was used. A Perkin Elmer UV–vis Spectrophotometer- Lambda was used to determine UV–visible spectra for the optical properties and controlled release studies.

## Results and discussion

### Powder X-ray diffraction

**Figure 1:**
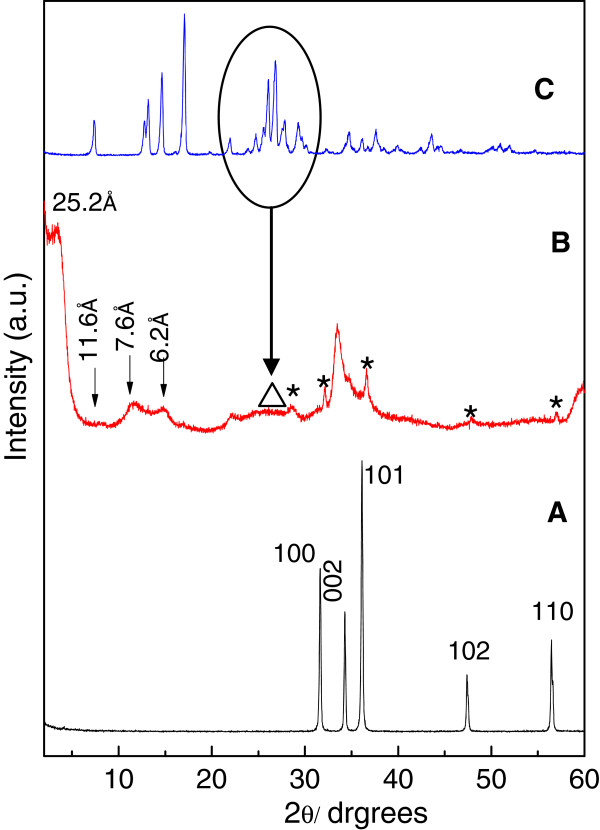
Powder X-ray diffraction results patterns for the zinc oxide (A), ASA-nanocomposite in which the symbol * represent zinc oxide peaks and triangle shows the adsorbed drug on the layers (B) and free 4-amino salicylic acid (C).

Figure 1A, B, and C shows the powder X-ray diffraction patterns for zinc oxide, 4-ASA-nanocomposite, and free 4-amino salicylic acid, respectively. As reported in the literature, the zinc layered hydroxide with nitrate as the counter anions can be prepared by adding the sodium hydroxide to zinc nitrate hexahydrate solution. This preparation give a sharp reflection peak with d-spacing, 9.74 Å at 2θ = 9.12 due to the 200 planes of the monoclinic structure [20].

Zinc oxide in aqueous solution formed ZLH layers and at the same time intercalate the anions between the layers, as was proposed earlier [21]. The layers of zinc hydroxide (Zn (OH) _2_) were formed on the surface of the solid particles due to hydrolyzed zinc oxide in an aqueous environment. The dissociation of Zn (OH) _2_ in a solution-solid interface produces Zn^2+^ species, which subsequently react with hydroxide group, drug anions and water in solution to form drug-zinc layered hydroxide compound. The Figure 1A shows five intense peaks between 30° and 60°, corresponding to diffractions due to the 100, 002, 101, 102, and 110 planes which characteristic of ZnO.The presence of a peak at a lower 2θ angle with d spacing of 25.2 Å indicates successful intercalation of the 4-amino salicylate anions into the interlayers of zinc-layered hydroxide (Figure 1B). The resulting 4-ASA nanocomposite sample contains a little amount of zinc oxide (indicated by a star). In addition, Figure 1B shows another three harmonics at 2θ = 7.6°, 11.7°, and 14.4°, with d values of 11.6, 7.6, and 6.2, respectively, resulting in an average basal spacing value of 24.0 Å. Some of the 4-amino salicylate anions are also adsorbed on the surface of zinc-layered hydroxide, which are indicated by triangles in the XRD diffractogram of the nanocompsite.

### Spatial orientation of intercalated 4-amino salicylic acid

**Figure 2:**
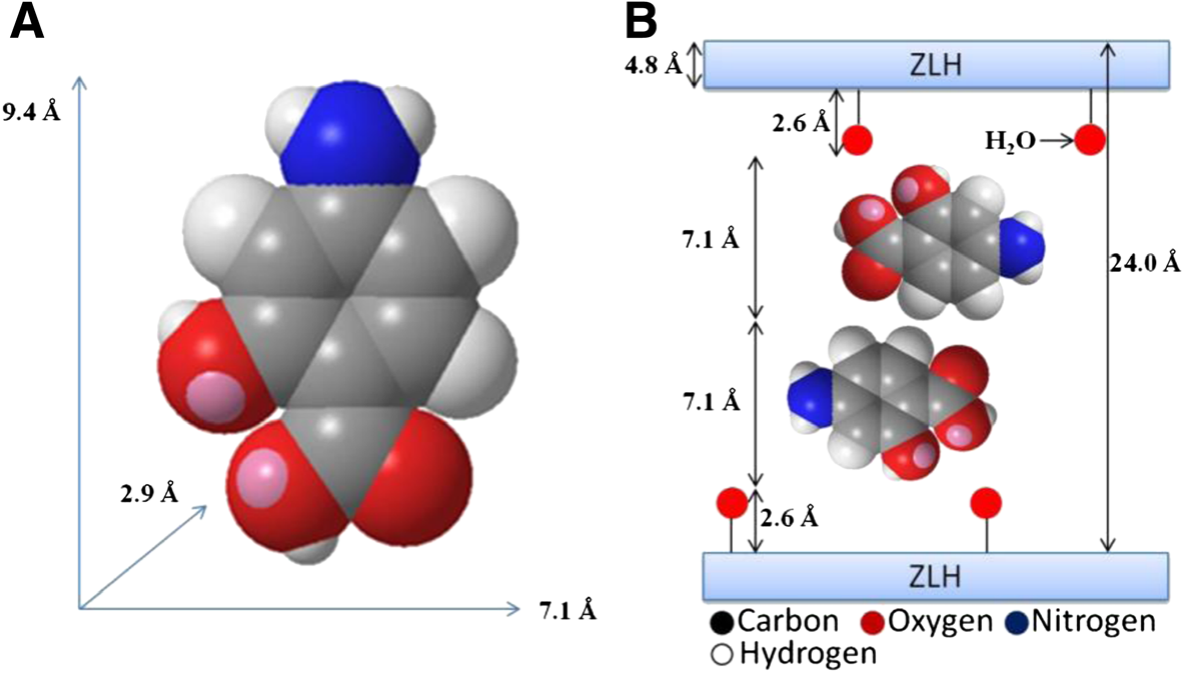
Spatial Orientation of 4-ASA three-dimensional structure of 4-amino salicylic acid (A) and molecular structural models of 4-amino salicylic acid intercalated between interlamellae of ZLH (B).

Figure 2A shows the three-dimensional molecular size of 4-amino salicylic acid obtained using a Chemoffice software 2008 (Cambridge, MA). The x, y and z axes of 4-amino salicylic acid was calculated and were found to be 9.4 Å, 7.1 Å and 2.9 Å, respectively. The X-ray diffraction pattern shows the average basal spacing, d = 24.0 Å for the 4ASA-nanocomposite. Taken into account that the thickness of the ZLH layer is 4.8 Å [19], and zinc tetrahedron has 2.6 Å thickness for each layer [22], therefore the gallery height of zinc layered hydroxide after the intercalation processes is 14.0 Å (24.0 – 4.8 + (2 × 2.6 Å)). The value is much similar to a double of the 7.1 Å axis (14.2 Å), which suggests that the anions are accommodated as an alternate bilayer.

### Infrared spectroscopy

**Figure 3:**
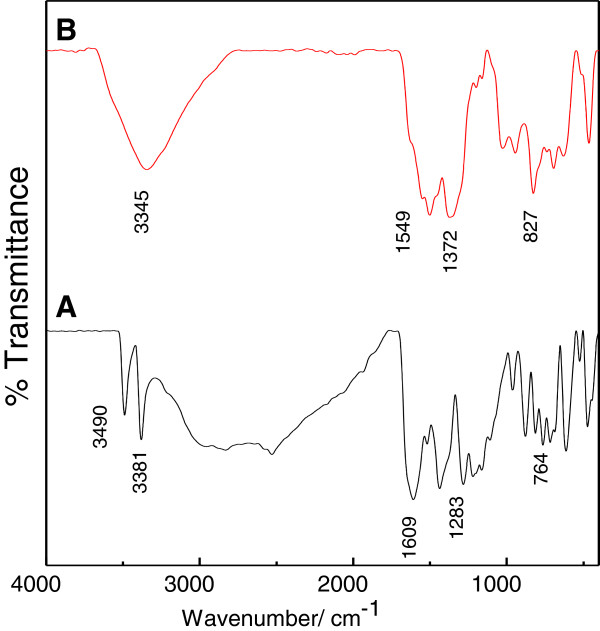
FTIR Spetra of free 4-amino salicylic acid (A) and 4ASA-nanocomposite (B).

The existence of free 4-amino salicylic acid (4-ASA) and the 4-ASA-ZLH-nanocomposite were further confirmed by FTIR study as shown in Figure 3A and B, respectively. The FTIR spectra of free 4-amino salicylic acid show two bands at 3490 and 3381 cm^-1^ due to the N–H asymmetric and symmetric stretching modes, respectively. The strongest absorption in the FTIR spectrum is observed at 1610 cm^-1^ which is due to the C=O stretching mode of COOH group. Others bands are listed in Table 1.

**Table 1:** **FTIR assignments for 4-amino salicylic acid and 4ASA-nanocomposite**[23,24]

**Assignments**	4-amino salicylic acid^*^	4ASA-nanocomposite^*^
**ν**_**as **_**(N-H)**	3490	Band for N-H symmetry and antisymmetry are overlapped with O-H stretching at 3345
**ν**_**s **_**(N-H)**	3381	
**ν (O-H) in the layer; H**_**2**_**O**	-	
**ν (C-C)**	1649, 1519	1502
**ν(C = O) in COOH**	1609, 764	-
**ν (O-H) ring**	3020,1283, 1163	1162
**δ**_**b **_**(CH), in plane bends**	1220, 1109, 813	1200, 1027, 827
**Stretching (C-H)**	813, 717	827, 738
**ν**_**as **_**(COO**^**-**^**)**	-	1549
**ν**_**s **_**(COO**^**-**^**)**	-	1372

^*^unit for the given number is the wavenumber (cm^-1^).

The disappearance of a C=O stretching band in the carboxylic group for free 4-amino salicylic acid after intercalation confirms that the species that were intercalated into the ZLH layers are in the anionic form of 4-amino salicylic acid, i.e. 4-amino salicylate. A band at 1549 cm^−1^ is due to the *v*_as_(COO^−^) mode, and another band at 1372 cm^−1^ is due to the symmetric vibration of *v*_s_ (COO^−^). These two bands indicate that there is an interaction between 4-amino salicylate and ZLH layers. The positions and relative intensities of the other bands almost coincide with those in the spectra of the free 4-amino salicylic acid. A bands due to Zn–OH translation modes are recorded below at 600 cm^−1^.

Table 1 FTIR assignments for 4-amino salicylic acid and 4ASA-nanocomposite [23,24].

### Elemental analysis

The elemental analysis (CHNS and ICP) of the product is usually used as a direct evidence to confirm the intercalation of the drug into ZLH inorganic interlayers. Table 2 shows that the C/N ratio for free 4-amino salicylic acid is 6.02 and this value is smaller than the C/N ratio of 4-ASA nanocomposite which is 6.8, indicating possible contamination by carbonate species during the preparation. Therefore, the loading of 4-amino salicylic acid between the ZLH was calculated using the nitrogen percentage, which gave 16.9%. The presence of the carbonate anions was confirmed by the presence of a broad peak at low 2θ (2θ =11. 65^o^) in the XRD patterns.

**Table 2:** Elemental chemical composition of free 4-amino salicylic acid and its nanocomposite

**Sample**	**C**^**a **^**(%w/w)**	**N**^**a **^**(%w/w)**	**C/N**	**Zn**^**b **^**(%w/w)**	**anion**^**a **^**(%w/w)**	**BET surface area m**^**2**^**/g**	**BJH pore volume cm**^**3**^**/g**	**BJH Average pore diameter Å**
ZnO	-	-	-	(80.30)	-	6.4	0.010	111
4-ASA	58.68	9.75	6.02	-	^-^	-	-	-
4-ASAN	10.54	1.54	6.84	52.7	16.86	80.5	0.260	96

Value in the parentheses is a theoretical value, ^an estimated^ from CHNS analysis, ^b^estimated from ICP analysis.

### Thermal analysis

**Figure 4:**
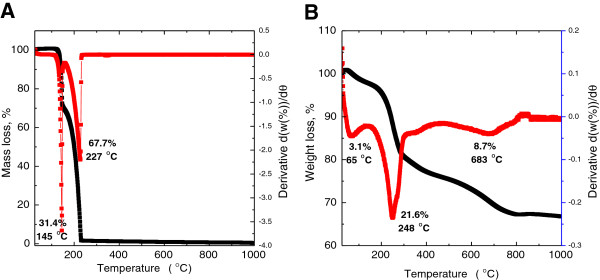
Thermograms of 4-ASA and 4ASA nanocomposite TGA-DTG thermograms of 4-amino salicylic acid (A) and 4ASA-nanocomposite (B).

The thermal behavior of 4-amino salicylic acid and 4ASA-nanocomposite was examined using thermogravimetric and differential thermogravimetric analyses (Figure 4). For 4-amino salicylic acid (Figure 4A), two main thermal events were clearly observed; the first one occurred in the region of 101–150°C, attributable to dehydration of 4-amino salicylic acid, corresponding to a sharp peak at 145°C with a 31.4% weight loss. The second stage at 151–235°C is due to thermal combustion of 4-amino salicylic acid [25], corresponding to a strong peak at 227°C with a 67.7% weight loss. Figure 4B shows that the thermal decomposition of 4ASA-nanocomposite progresses through three major stages of weight loss, occurring at temperature maxima of 65°C, 248°C and 683°C with weight losses of 3.1%, 21.6% and 8.7%, respectively. The first stage of weight loss in the range of 39–138°C is due to the removal of physisorbed water on the external surface of the layered double hydroxide. The second weight loss is mainly due to combustion of 4-amino salicylic acid anions at 248°C. Increase in the decomposition temperature of 4-amino salicylic acid from 227°C (for free 4-amino salicylic acid) to 248°C (for 4-amino salicylic acid intercalated into the nanocomposite) indicates better thermal stability of 4-amino salicylic acid in the intercalated form than the free 4-amino salicylic acid. This presumably is due to electrostatic attraction between the negatively charged functional group of 4-amino salicylic acid and the positively charged zinc layered hydroxide.

### Surface properties

**Figure 5:**
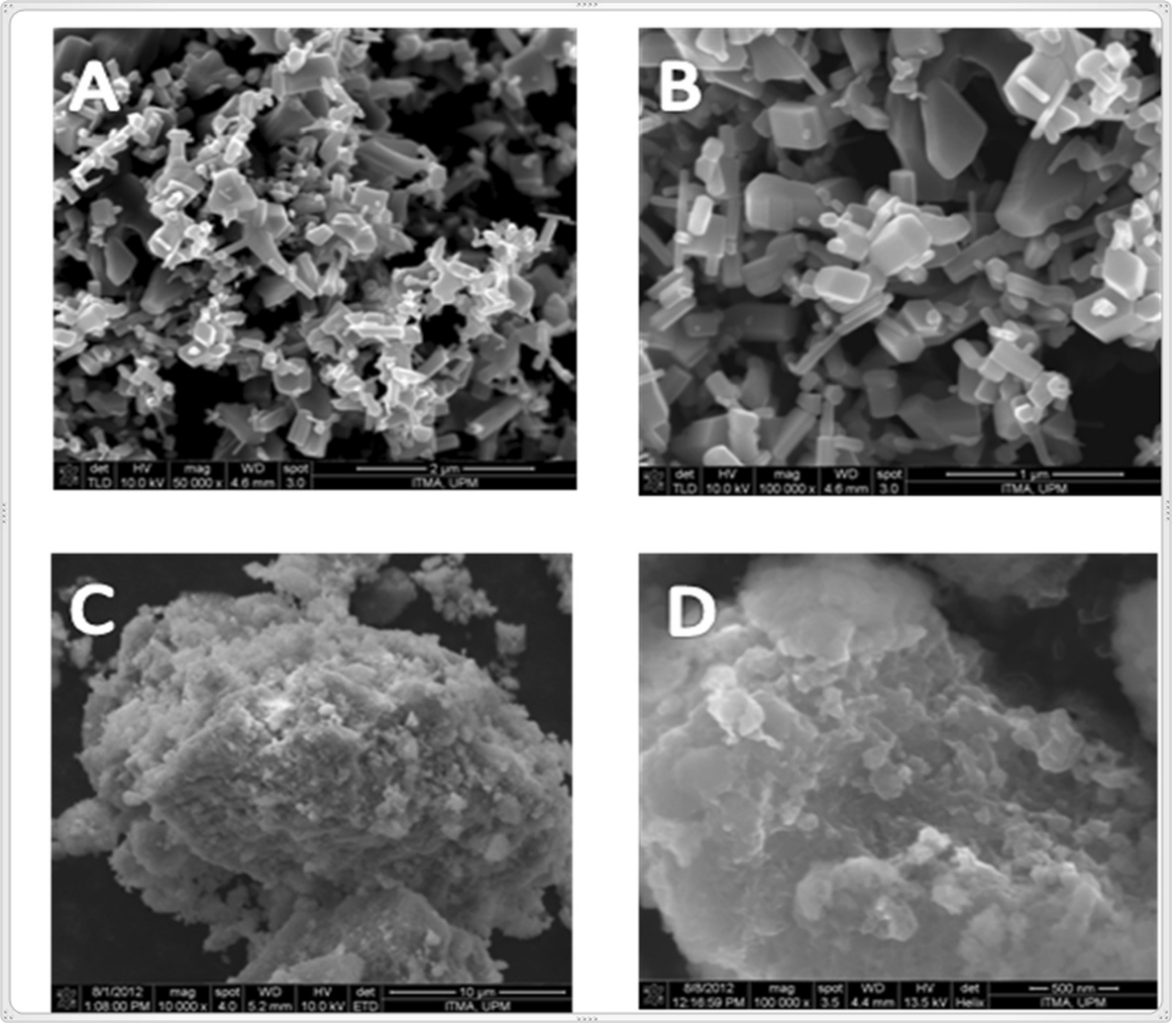
FESEM Micrographs of ZnO and 4-ASA nanocomposite field emission scanning electron micrographs of zinc oxide (A and B), and 4-ASA nanocomposite (C and D).

Figure 5 A-D show the morphology of starting material, zinc oxide and its ZLH-4-ASA nanocomposite obtained by FESEM. Zinc oxide shows a nonuniform granular composition without any specific shape, and with various sizes (Figure 5A and 5B). As shown in the Figure 5C and 5D, there is a significant difference in the morphology of the both samples; the 4-ASA nanocomposite shows agglomerates of compact and non-porous structure which indicates the transformation of the zinc oxide to the zinc layered hydroxide nancomposites. This result is very similar to the morphology of other intercalated compounds such as hippurate-zinc layered hydroxide and hippurate-Zn/Al-LDH [26].

**Figure 6:**
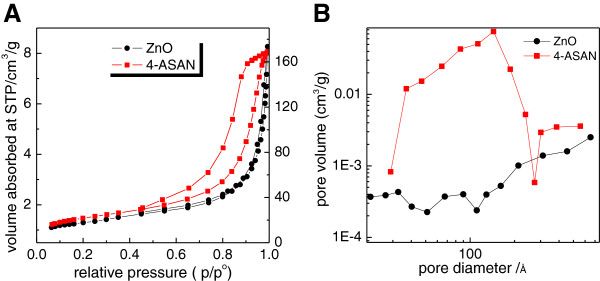
**Control Release Profiles I) Release profiles of a physical mixture of 4-amino salicylic acid with pristine zinc layered hydroxide, (II) Release profiles of 4-amino salicylic acid from the 4-ASA nanocomposite at pH 7.4 (A) and pH 4.8 (B).** Inset shows the release profiles of 4-amino salicylic acid from the nanocomposite at pH 4.8.

Figure 6 shows the nitrogen adsorption-desorption isotherms of zinc oxide and 4-ASA nanocomposite. All the adsorption isotherms are of Type IV according to IUPAC classification, indicating mesoporous type material (20–500 Å) [27].The volume adsorb increases slowly at a low relative pressure in a range of 0.0-0.8, followed by a rapid adsorption at a relative pressure more than 0.8. For the 4-ASA nanocomposite, the optimum uptake is slightly higher than their corresponding starting material, zinc oxide. The desorption branch of the hysteresis loop for 4-ASA nanocomposite is wider than zinc oxide, indicating different pore texture of the resulting materials. This is due to modification of the pores as a result of the intercalation process and formation of the new nanocomposite [28]. Table 2 shows the BET surface area of the zinc oxide and 4-ASA nanocomposite. As shown in Table 2, the surface area of the resulting nanocomposite is generally higher than their corresponding zinc oxide. The surface area of zinc oxide and 4-ASA nanocomposite is 6.4 and 80.5 m^2^/g, respectively.

The BJH pore size distributions for zinc oxide and 4-ASA nanocomposite are shown in Figure 6B. All the materials are of a mesoporous-type. The BJH pore size distribution for 4-ASA nanocomposite shows an intense peak at around 110 Å, compared to zinc oxide, which show less intense broad peaks at around 10 and 90 Å. The difference in peak values and pore volume values in the pore size distribution of 4-ASA nanocomposite and zinc oxide indicate the modification of the pure texture which occurred when intercalation of 4-amino salicylic acid took place, in agreement with the formation of the nanocomposite. The BJH average pore diameter and pore volume are shown in Table 2.

### Release behavior of 4-amino salicylic acid from 4-ASA nanocomposite

**Figure 7:**
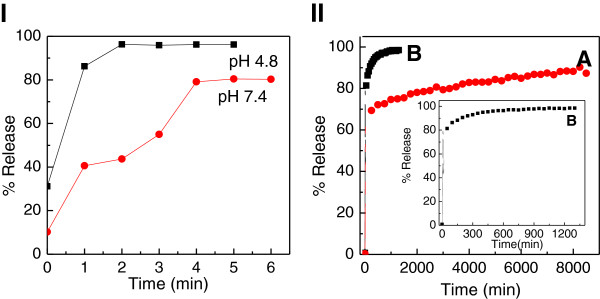
Surface area analysis isotherms Adsorption-desorption isotherms (A) and BJH pore size distribution (B) for zinc oxide and 4-ASA nanocomposite.

Figure 7 (I) shows a release of 4-amino salicylic acid from its physical mixture in both PBS solutions at pH 7.4 and 4.8. Generally, 4-amino salicylic acid was immediately released within the first five minutes, and reached at 80% and 97%, at pH 7.4 and 4.8, respectively.

The release profiles of 4-amino salicylic acid from the 4-ASA nanocomposite on exposure to either a pH 4.8 or pH 7.4 environments are shown in Figure 7 (II). The release rate of 4-amino salicylic acid from the nanocomposite is obviously depending on pH; release at pH 7.4 is remarkably slower than that at pH 4.8. The percentage release of 4-amino salicylic acid from the nanocomposite reaches about 100% within about 1330 minutes when exposed to pH 4.8 compared to only about 92% within about 8540 minutes at pH 7.4. The difference in the release rate may be due to possible differences in mechanism [29,30]. At pH 7.4, ZLH layers are more stable compared to pH 4.8. This indicates that the ZLH layers in acidic media (pH 4.8) dissolve more easily and the release occurs through both the dissolution of ZLH layers and the diffusion. On the other hand at pH 7.4 media, the release occurs through a diffusion mechanism.

The release rate of 4-amino salicylic acid from its nanocomposite was obviously very much slower than that from the physical mixture, indicating that the nanocomposite potentially can be used as a better alternative as compared to the free drug.

### Release kinetics of 4-amino salicylic acid from 4-ASA nanocomposite

Usually, the release process of drug molecules from the nanocomposite may be described using pseudo-first order, pseudo-second order or parabolic diffusion kinetic equations.

Pseudo-first-order kinetic equation may be represented in the linear form as [31]

$$\ln\left(q_{e}{-q}_{t}\right){=\ln\;q}_{e}{–k}_{1}t$$

where q_*e*_ and q_*t*_ are the equilibrium release amount and the release amount at any time (*t*), respectively. The *k*_1_ value can be obtained from the slope by plot ln(*q*_e_ − *q*_t_) against *t*.

Pseudo-second-order kinetic equation may be represented in the linear form as [32],

$${t/q}_{t}{=1/k}_{2}q_{e}{}^{2}{+t/q}_{e}$$

The parabolic diffusion kinetic equation may be represented as [33],

$$\left({1-M}_{t}/M_{o}\right){/t=k\;t}^{-0.5}+b$$

where M_o_ and M_t_ are the drug content remained in the ZLH at release time 0 and t, respectively.

**Figure 8:**
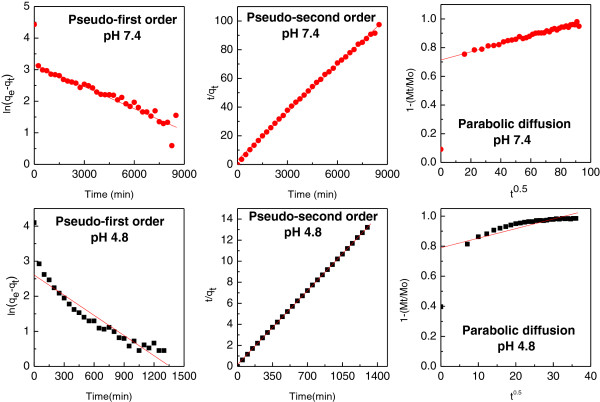
**Cytotoxicity analysis cell viability (MTT assay) of 3 T3 exposed to various gradient concentrations at 24, 48 and 72 hours.** The data presented are mean ± SD of triplicate values.

Using the above equations, it was found that the pseudo-second order model is more satisfactory to describe the release kinetic process of 4-amino salicylic acid from 4-ASA nanocomposite. Figure 8 shows plots of the fitting of 4-amino salicylic acid released from 4-ASA nanocomposite. At pH 4.8, the correlation coefficient (R^2^) and *k*_2_ values are 0.9999 and 1.0 × 10^-4^ mg/min, respectively and at pH 7.4, the corresponding value is 0.9982 and 3.9 × 10^-5^ mg/min, respectively. The release kinetic results are similar to the hippurate-zinc layered hydroxide, ellagic acid-zinc layered hydroxide and citirizine-zinc layered hydroxide synthesized by the direct reaction of zinc oxide with drugs [14,15,33] (Table 3).

**Table 3:** **Correlation coefficient (R**^**2**^**), and rate constants (k) obtained by fitting the data of 4-amino salicylic acid release from 4-ASA nanocomposite into PBS solution at pH 4.8 and 7.4**

**pH**	**Saturation release (%)**	**R**^**2**^			**pseudo-second order**
		**pseudo-first order**	**pseudo-second order**	**Parabolic diffusion model**	**rate constant (k) (mg/min)**
7.4	92	0.9339	0.9982	0.8961	3.9 × 10^-5^
4.8	100	0.8839	0.9999	0.5925	1.0 × 10^-4^

### Cytotoxicity analysis of 4-amino salicylic acid (4-ASA) and 4-ASA nanocomposite

**Figure 9:**
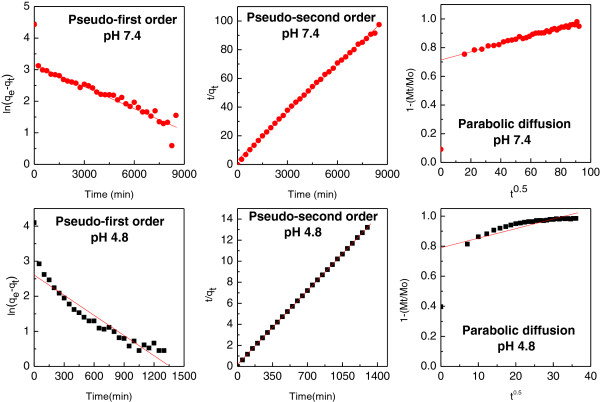
Kinetics of control release fitting the data of 4-amino salicylic acid release from 4-ASA nanocomposite into PBS solutions at pH 7.4 and 4.8 using pseudo-first, pseudo-second order kinetics and parabolic diffusion model.

Figure 9 shows the effect of free 4-amino salicylic acid, 4-ASA nanocomposite and ZLH on cytotoxicity of 3T3 mouse fibroblast cells at different gradient concentrations; 0.781, 1.562, 3.125, 6.25, 12.5, 25 and 50 μg/mL and various time points; 24, 48 and 72 hours using MTT assay. ZLH at concentrations from 0.781 to 12.5 μg/mL did not show any reduction in cell viability at 24 and 48 hours duration of inhibition, but exposure with the same concentration for 72 hours shows significant cytotoxicity. Cells exposed to 25 and 50 μg/mL of ZnO NPs for 48 hours showed 50% and > 90% reduction, respectively in cells viability (Figure 9B) but not in 24 hours exposure of nanocomposite (Figure 9A). In addition, 72 hours inhibition with ZLH nanocomposites, shows stronger cytotoxicity when compared with 24 and 48 hours.

## Conclusion

A nanocomposite of 4-amino salicylic-ZLH was obtained by the intercalation of 4-amino salicylic acid anions into zinc layered hydroxide using zinc oxide as starting material. XRD technique was used to confirm the intercalation 4-ASA. The resulting nanocomposite has a basal spacing of 24.0 Å, indicates that the guest adopted a bilayered arrangement. FTIR study shows the presence of functional groups of both 4-amino salicylic acid anions and inorganic host. The thermal stability of intercalated 4-amino salicylic acid anions were enhanced remarkably compared to the pristine 4-amino salicylic acid anions whose decomposition occurs at 227°C. The loading of 4-aminosalicylic acid in the nanocomposite was estimated to be about 16.9% (w/w). The release of 4-amino salicylic acid anions from nanocomposite was found to occur in a controlled manner, so the resulting material is suitable for a controlled-release formulation. Based on preliminary cytotoxicity studies of the nanocomposite, more detailed investigations are needed to elucidate the specific cellular mechanism of toxicity. Further investigations on a broader set of specific cell lines combined with an evaluation of the detailed cellular mechanism of nanocomposite cell interactions are also important. This investigation helps to establish the suitability of pre-clinical model for early toxicity assessment of the nanocomposite. The work on the biocompatible sustained release formulation is a step towards reduction in dosing frequency of the drugs and subsequently enhances the patient compliance and make the tuberculosis chemotherapy patient-friendly.

## Competing interests

The authors declare that they have no competing interests.

## Authors’ contribution

BS, MZH and SHH-Al-Ali carried out all the chemistry work including preparation, characterization and interpretation and write of manuscript. While the authors PA and SF carried out all the biological work and its interpretation and write up of the biological part in the manuscript. All authors read and approved the final manuscript.
